# Enhancing engagement beyond the conference walls: analysis of Twitter use at #ICPIC2019 infection prevention and control conference

**DOI:** 10.1186/s13756-021-00891-1

**Published:** 2021-01-25

**Authors:** Romain Martischang, Ermira Tartari, Claire Kilpatrick, Graham Mackenzie, Vanessa Carter, Enrique Castro-Sánchez, Hilda Márquez-Villarreal, Jonathan A. Otter, Eli Perencevich, Denise Silber, Julie Storr, Jason Tetro, Andreas Voss, Didier Pittet

**Affiliations:** 1 Infection Control Programme and WHO Collaborating Centre on Patient Safety, University of Geneva Hospitals and Faculty of Medicine, 4 Rue Gabrielle-Perret-Gentil, 1211 Geneva, Switzerland; 2 Institute of Global Health, Faculty of Medicine, University of Geneva, Geneva, Switzerland; 3 Faculty of Health Sciences, University of Malta, Msida, Malta; 4 S3 Global, Glasgow, UK; 5 NHS Education for Scotland, Edinburgh, UK; 6 Healthcare Communications, Social Media, Cape Town, South Africa; 7 Stanford University Medicine X ePatient Scholar Program, Stanford, CA USA; 8 NIHR Health Protection Research Unit (HPRU) in HCAIs and AMR at Imperial College London, and Imperial College Healthcare NHS Trust, Infection Prevention and Control, London, UK; 9 Department of Public Health, University of Guadalajara, Jalisco, Mexico; 10 Center for Access & Delivery Research & Evaluation (CADRE), Iowa City Veterans Affairs Health Care System, Department of Internal Medicine, University of Iowa Carver College of Medicine, Iowa City, IA USA; 11 Basil Strategies, Paris, France; 12 Infection Prevention and Control Consultant, Edmonton, AB Canada; 13 Department of Medical Microbiology, Radboud University Medical Center, Nijmegen, The Netherlands; 14 Department of Clinical Microbiology and Infectious Diseases, Canisius-Wilhelmina Hospital, Nijmegen, The Netherlands; 15 REshape Center for Innovation, Radboud University Medical Center, Nijmegen, The Netherlands

**Keywords:** Social media, Social networking, Science communication, Medtweeter, Tweeter, Twitter, Infection prevention and control, Public, Patient, Patient participation, Medical conference

## Abstract

**Background:**

Social media may provide a tool, when coupled with a patient-included™ conference, to enhance the engagement among the general public. We describe authors and potential readers of Twitter content surrounding a patient-included™ scientific congress, the International Consortium for Prevention and Infection Control (ICPIC) 2019.

**Methods:**

Retrospective observational analysis of Twitter users posting with the #ICPIC2019 hashtag during the conference. Tweet authors, overall followers, and active followers were categorized according to their Twitter biographies using unsupervised learning. Diversity of professional backgrounds of Tweet authors and their followers was explored. Network analysis explored connectedness between the reach of authors.

**Results:**

In total, 1264 participants attended ICPIC 2019, of which 28 were patients. From September 7 to 16, 2019, we were able to categorize 235′620 (41%) followers linked to 474 (76%) authors. Among authors and followers, respectively 34% and 14% were healthcare workers, 11% and 15% were from industry representatives, 8% and 7% were academic researchers. On average, 23% (range 9–39%) followers belonged to the same categories as authors. Among all followers categorized, only 582/235 620 (0.25%) interacted with original messages, including healthcare workers (37%), global and public health (12%), academic research (11%) and those from industry (11%). Though the similarity between Tweet authors and followers was supported by network analysis, we also observed that non-healthcare workers (including patients) appeared to have more diverse followers.

**Conclusions:**

We observed the participation of numerous Tweet authors and followers from diverse professional backgrounds potentially supporting the benefit of including patients in conferences to reach a more general, non-specialized public.

## Background

Most international conferences, including those on infection prevention and control (IPC) and infectious diseases remain scarcely accessible to an extensive set of attendees for multiple reasons (time, budget, country entry requirements, etc.). Involvement of all stakeholders, including patient and public involvement is considered critical indeed to bend the curve on the rising global and economic tide posed by antimicrobial resistance (AMR), as one example related to infections [1]. Improving communication from scientific content delivered during the conference to the scientific community but also the general public might be essential to reach the aforementioned objective.

Twitter provides a unique opportunity to bridge the divide for researchers, patient communities and the public to engage with scientific information remotely in a more accessible, inclusive, and diverse platform keeping up with cutting-edge research, sharing knowledge, and having the opportunity to learn [2]. Interactions with published messages include tweets, retweets which share original messages and quote tweets which include personal comments, and replies related to the original tweet. These interactions are unilateral, meaning that followers are not always followed. More recently, Twitter has reshaped the impact of scientific conferences by engaging virtual followers as documented across medical specialities [3–6] including infectious diseases and IPC [7–9].

Studies have identified the importance of including patients as partners in scientific conferences, helping to direct research and current discussion in a patient-centric approach, driving the future of healthcare [10, 11].

The 5^th^ international consortium for prevention and infection control (ICPIC) [12], is an established 4-day congress in the prevention of healthcare-associated infections and control of antimicrobial resistance that is held biannually. ICPIC2019 was the first in IPC conferences to integrate patient participation and conferred a patient-included™ charter status [13] (Additional file 1: Table 1). A conference successfully meeting all five of the charter’s pillars namely: (1) codesign (patients participate in the selection of topics and speakers), (2) engagement (including patients as presenters and in the audience), (3) accommodation (support in travel and accommodation and provide scholarship), (4) disability requirements (accommodating the physical needs of patients) and 5) virtual participation (free online video streaming) may be accredited as a Patients Included™ event [13]. Patient integration in IPC conferences is an important step to bring patients closer to the conversations driving patient safety and to ultimately improve the lives of patients and their families [12]. Inclusion and active engagement of patients as stakeholders can help drive knowledge dissemination and identify issues that matter most to patients, caregivers and their families (Table 1).

**Table 1:** Labels defining the 14 clusters based on Twitter profiles

Final labels	Detail	Comments^a^
Others	Advocates and politics	Biographies mainly expressing a stance for certain causes (racial, gender, politics, environmental…)
	Characters	Non-English biographies or including special characters (that have not been filtered out)
	Hobbies, families & life balance, spirituality^b^	Biographies around personal interests with a strong focus on families, religion and hobbies
	Time and place	Biographies mainly containing geo-temporal information, indicative for event, gatherings, conferences, congress, tourism…
Advertising	Advertising	Biographies suggestive for publicity, advertisements
Fintech–digital marketing	Fintech–digital marketing	Biographies suggestive of using novel technologies in innovation and private industries. More specific for fintech (bitcoin, cloud, blockchain), and digital marketing (social media, marketing)
Industries	Industries and manufacturers	Biographies representing industries delivering a product
	Industry-related services	Biographies expressing a service often targeting industries. In this dataset, it has a strong focus on health care related industries, but also include human resources, consulting, education providers…
Media	Media and music	Biographies related to audio-visual content, including authors, publishers, bloggers, editors…
Patient support, foundation, advocacy and alternative therapies	Patient support, foundation, advocacy and alternative therapies	Biographies oriented to patient’s health, mainly using popular wording for diseases and health (disease, life, pain, chronic disease). Gather disease’s survivors, foundations or associations’ oriented to patient’s care, but also alternative therapies and caregivers
Clinical leaders and Healthcare Workers	Physicians	Biographies expressing specialized medical wording, also gather medical organizations
	Clinical leaders and healthcare workers—healthcare quality improvement	Biographies expressing a will of healthcare quality improvement, with certain specializations or belonging to certain society/organizations
Academic research	Academic research	Biographies expressing wording specifics for academic research such as degrees. Has a strong focus on biology and microbiology (genomic, biology, bioinformatics)
Public and global health	Public and global health	Biographies expressing research or interests in public health concerns (sustainability, child care, equity, justice, climate)

^a^Words might be represented in multiple labels. What allows the discrimination between these labels is the specific distribution of all words presents in biographies

^b^2 clusters were merged, including “hobbies, families” and “life balance, spirituality”

Twitter may enhance the experience of scientific congresses to a wider audience and generate international engagement and global reach [14, 15]. However, this is not a guarantee for various reasons, such as the number of followers [15], and the content of published messages that need to be informative and of interest to non-attending individuals in order to sustain engagement [16]. Furthermore, an echoing effect has been observed with scientists mainly reaching other scientists, impacting the spread of the message to other stakeholders [15]. Assessing this echoing effect might estimate the spread of content from scientific conferences among the general public. Through non-supervised clustering approach based on biographies of the Twitter participants and their followers, we might describe more in detail the categories of stakeholders involved in the spread of online content [17–21]. As patients’ status might be hardly ascertained based on biographies, such analysis would focus on the diversity of categories of Twitter users observed, hypothesizing that they represent past, present and future patients.

This study was performed: (i) to assess how ICPIC2019 allowed conference participants to reach out to other peers (in-reach) and to non-scientific audiences (general public) (outreach) through Twitter discussion; (ii) to compare the professional background of followers of participants (“reach”), and followers that interacted with original tweets; (iii) to explore connectedness between followers of each participant and estimate the potential spread of scientific information.

## Material and methods

### Study design and objectives

We conducted a retrospective observational study of social media data (tweets, retweets, mentions, digital impressions) covering a total of nine days Twitter activity (from September 7 to 16, 2019) during the ICPIC patient-included™ scientific congress (September 10–13, 2019) [12]. During this period, all tweets with the official hashtag of the congress #ICPIC2019 were extracted, including original tweets, retweets, quotes, and replies. Information on the users (defined as Tweet author here), as well as the followers of the authors (reach), was extracted.

An analysis of the digital impressions among the professional background categories of authors and their followers was conducted, including the diversity of followers among specific categories of authors, the diversity of followers that interacted with original Tweet messages concerning the scientific conference, and the connectedness between followers of each participant. Authors were defined as users who published an original message, a retweet, a quote, or a reply, including the hashtag #ICPIC2019 during the study period. Reach was defined as all the followers of these authors. Active reach were the followers that interacted with the original tweet message using quote, retweet or reply. Ethics approval was requested and waived by the IRB committee in Geneva, Switzerland.

### Data extraction and pre-processing of Twitter profiles

### Latent Dirichlet allocation (LDA)

Topic modelling with the unsupervised clustering method named “Latent Dirichlet Allocation” has been used in multiple fields to clustering information from social media [17–20], and Twitter users together, based on their biographies [21]. In brief, the LDA is a Bayesian method estimating the probability of words belonging to a topic (beta probabilities), and the probability of topic belonging to a biography (gamma probabilities). More information on this method is detailed in the appendix (Additional file 1).

### Cluster labelling

After estimation of gamma and beta probabilities, reviewing of the biographies with the highest probability to belong to each topic, and reviewing of the words most likely associated with each topic, it was necessary to define a label for each cluster. Labels were defined by two blinded researchers (RM and ET) based on the 30 biographies with the highest gamma probability and the 20 words with the highest beta probability for each cluster. For further help, word clouds of the 50 most frequent words from biographies in each cluster were computed. Discordancies were resolved by consensus. These labels were then validated on a naive dataset (not used during the definition of labels), including five documents randomly extracted per four categories of gamma probabilities (30–50;51–60;61–80;81–100%) for all clusters. This even representation of biographies within a range of gamma probabilities helped to define a threshold of gamma proportion to ascertain a topic to a biography. Biographies previously used to define the label were not validated. In case of doubt, during the validation of these labels, the professional background of the authors was manually searched through the Internet.

### Comparison of the diversity in followers

Only topics with the highest gamma probability were retained because these were most likely to accurately categorize authors and followers. Then followers of different categories of authors were compared. Twitter users with a professional background estimated based on their category were selected (by increasing the probability to belong to these clusters) to compare the diversity of their respective followers. Network analysis was used to visualize the relationship between different categories of authors and their followers.

### Active followers

To estimate the reach of original tweets (active reach), users who retweeted, quoted, or replied to an original tweet were extracted to determine the number of “active followers”. Active followers, considered initially as author users because of the content they generated, will be considered as followers in this analysis. The proportion of active followers was then stratified among the different categories. Network analysis stratified by the type of interaction was also used to visualize the different actors and their respective categories.

Data extraction through Twitter Application Programming Interface, data mining, Latent Dirichlet Allocation, and Network analysis were performed using R to provide estimates of connectedness between authors and followers and according to their respective predicted categories. RStudio (v.3.6.0.) and RAnalyticFlow (v.3.0) were used with the following packages (rtweet, gggraph, iggraph, tidytext, topicmodels, tm, SnowballC, and stopwords) (R Foundation for Statistical Computing, Vienna, Austria; 2017; https://www.R-project.org/).

## Results

In September 2019 (ICPIC2019), a total of 1264 participants attended the conference of which 28 were patients.

### Data extraction of followers

**Fig. 1:**
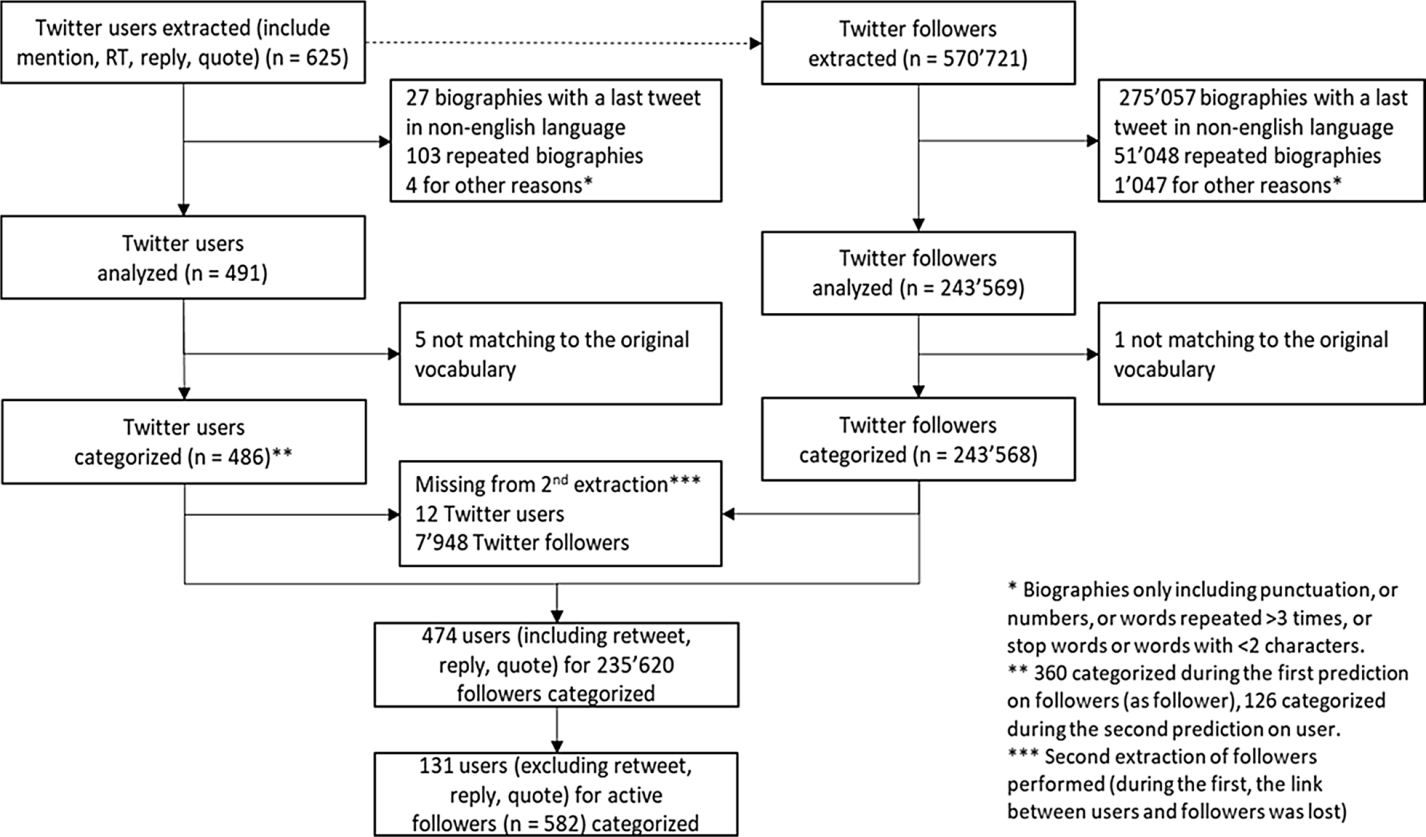
Flowchart of authors and their followers

A total of 3′561 tweets from 625 Twitter authors, as well as information on 570′721 unique followers, were extracted. Authors and followers were excluded if their last tweet was not in English, in case of duplicate biographies, and for other reasons (Fig. 1). Two data extractions were necessary, with minor information loss in between. In total, 235′620 (41%) followers linked to 474 (76%) authors were categorized (Fig. 1). Among authors and followers categorized, authors had a median number of followers of 229 (IQR 63–790). English was used among authors and followers, 86% and 52% of the time (Additional file 1: Fig. 1). Biographies of authors and followers included respectively 10 (IQR 6.5–12.5), and 10 (6–13) words per biography, and 6 (4–8), and 6 (5–7) characters per word. Words expressed in the biographies of followers were mostly related to health (Additional file 1: Fig. 2 & 3).

**Fig. 2:**
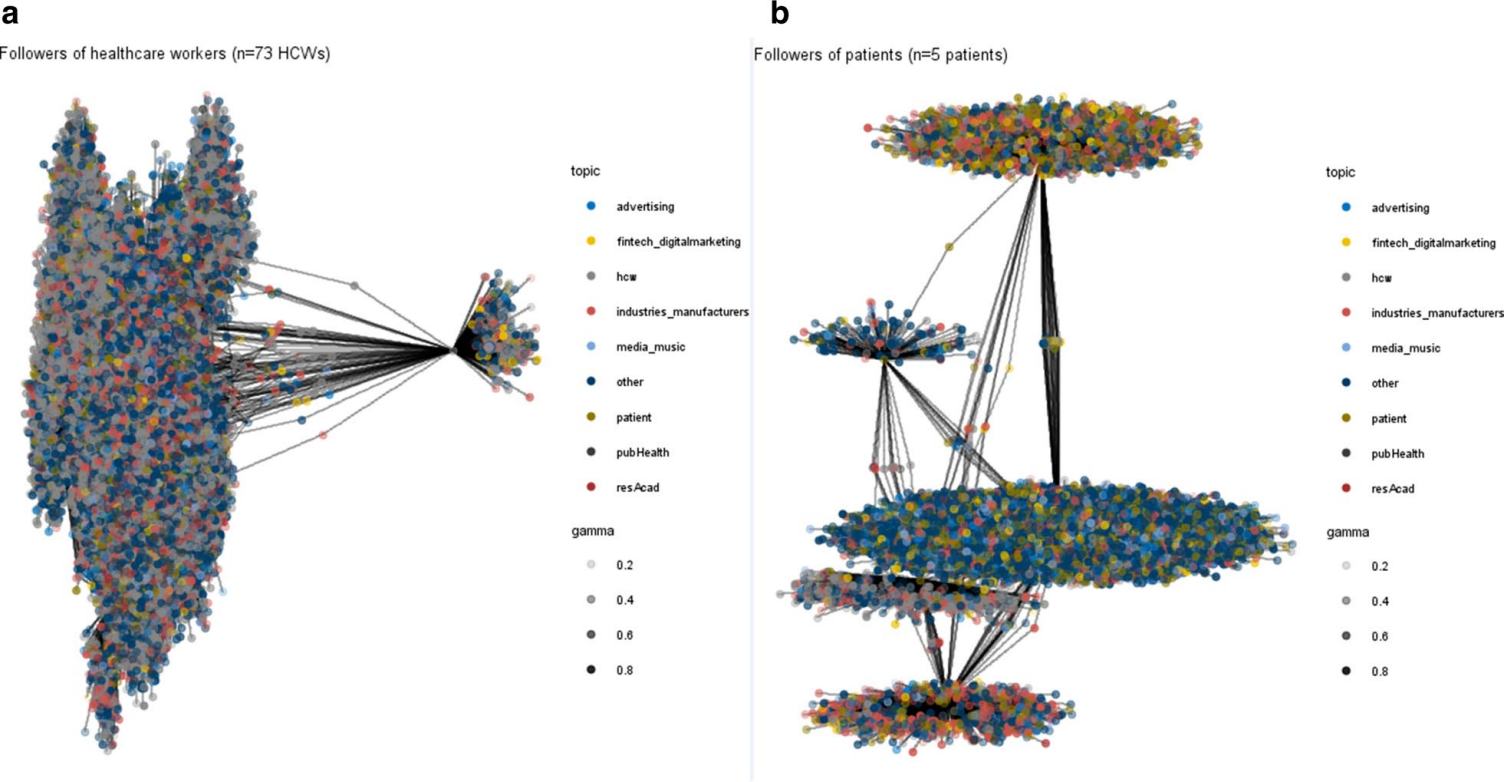
**a** Network analysis of followers from healthcare workers (authors with gamma > 0.5). **b** Network analysis of followers from patient-oriented biographies (authors with gamma > 0.4)

**Fig. 3:**
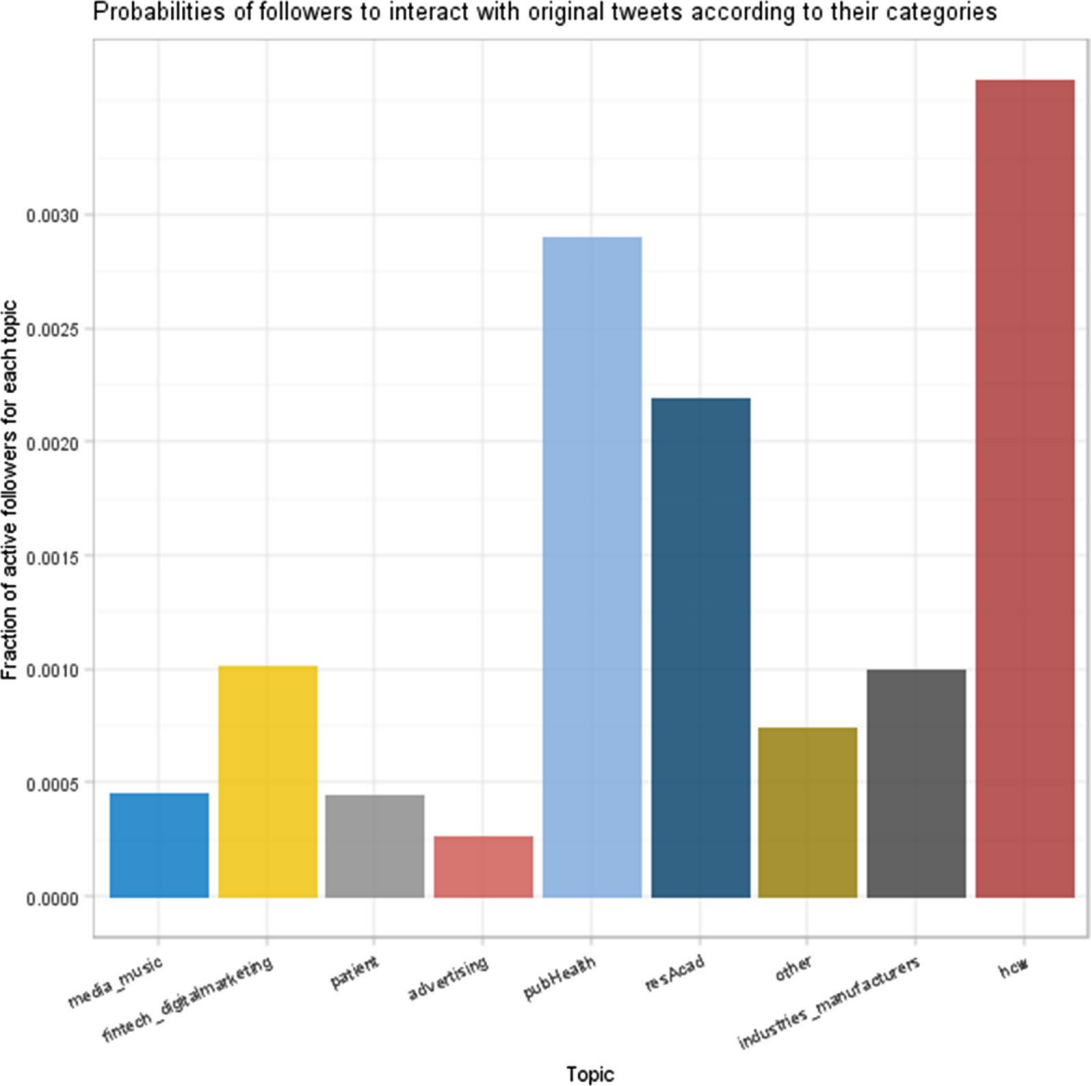
Proportion of active followers among total followers for each category

### Latent Dirichlet allocation

Fifteen categories of Twitter biographies were created and investigated (Additional file 1: Fig. 4, 5 & 6). These categories were labelled based on exploring the documents and words extracted, as well as calculated word clouds (Additional file 1: Table 1; Additional file 1: Fig, 7). After merging different categories, we got in total 9 clusters labelled: “Clinical leaders and healthcare workers”, “Industries”, “Others”, “Fintech & Digital Marketing”, “Media and Music”, “Advertising”, “Patient support, Foundation, Advocacy and Alternative Therapies”, “Public and global health”, and “Academic research”. (Additional file 1: Table 2).

**Fig. 4:**
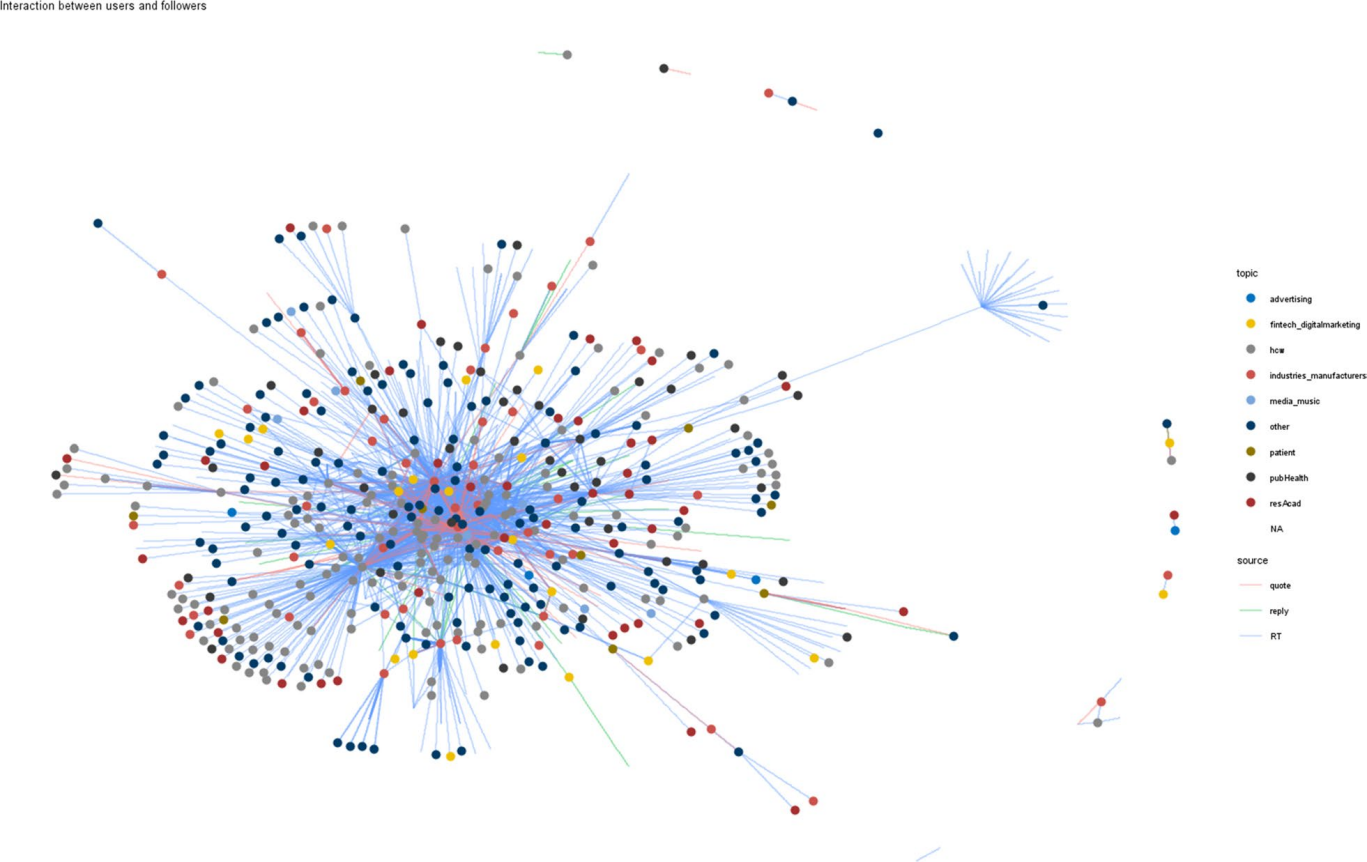
Nature of interactions between authors and their followers

During the validation of these labels, 81.8% of the agreement was reached between the two researchers, and overall performance of the label was 90% when assessing the real background of the author (Additional file 1: Table 2). Though variable discriminating performance across the topics, after repetition of the validation process over a range of probabilities to assess a topic, a cut-off at 40% seemed an adequate compromise to retain most represented categories for each author and follower (Additional file 1: Table 3 and Additional file 1). We filtered out all categories with a probability below 40%, reducing this number to 363 (58%) and 181′192 (32%) of authors and followers respectively. Predominant clusters among followers were “others”, representing 40% of all followers’ biographies, followed by those from industry (15%) and healthcare workers (14%). Among authors, 33% were classified as “others”, while 34% were healthcare workers, 11% industries, and 8% of academic researchers (Additional file 1: Table 4 & 5). Distribution of gamma proportions was similar among clusters for followers and authors with a mean of 49–65%.

#### Exploration of categories of followers based from defined authors

To obtain a reliable sample of Twitter authors in each category, we filtered out all authors with a gamma probability below 50% and compared their relative distribution of followers. Among 355 Twitter authors and their 153′726 followers remaining, the proportion of followers categories was significantly dependent on the authors’ categories (Table 2). On average, 22.9% (9–39%) of followers belonged to the same categories of authors. These variations in the diversity of followers when considering each authors’ category were supported by network analysis, observing much more diversity in followers of patients compared to followers of healthcare workers (Fig. 2a, b, Additional file 1: Fig. 8–11).

**Table 2:** Followers of different authors’ categories

		Categories of authors selected (gamma > 50%, n = 355)							
		1 (n = 1)	2 (n = 6)	3 (n = 2)	4 (n = 1)	5 (n = 17)	6 (n = 18)	8 (n = 27)	9 (n = 73)
Categories of followers	1	**11%**	4%	7%	5%	3%	3%	3%	2%
	2	8%	*20%*	4%	**19%**	4%	2%	6%	2%
	3	5%	9%	**12%**	1%	4%	3%	6%	3%
	4	7%	5%	6%	9%	2%	2%	4%	2%
	5	6%	3%	4%	5%	*28%*	7%	5%	6%
	6	8%	1%	2%	6%	7%	*36%*	4%	7%
	8	**14%**	*23%*	9%	**15%**	**12%**	9%	*28%*	**13%**
	9	7%	**10%**	9%	8%	**17%**	**13%**	**15%**	*39%*

Topic 1: Media, Topic 2: Fintech-digital marketing, Topic 3: Patient oriented, Topic 4: Advertising, Topic 5: Global and Public health, Topic 6: Academic research, Topic 7: Others, Topic 8: Industries, Topic 9: Healthcare workers

### Active followers of authors

Authors who retweeted, quoted, or replied to an original tweet were defined as “active followers”. In total, 582 active followers interacted with original tweets from 131 authors. These interactions were 561 retweets, 56 quotes and 40 replies. 338 (58%) of these followers were categorized. The majority of followers who interacted with original tweets were: healthcare workers (37%), global and public health (12%), academic research (11%) and industries (11%) (Figs. 3, 4). The proportion of active followers among the total reach was low (Additional file 1: Table 6), but was still the highest for healthcare workers and public health professionals.

## Discussion

Our study used unsupervised learning in the tweets mentioning #ICPIC2019 for profiling of both authors and their respective followers according to their biographies, in the context of a patient-included™ conference. Including only English Tweets (based on their last tweet), the volume of followers and authors categorized was significant, with 235′620 followers linked to 474 authors. Unsurprisingly, we observe that the majority of Twitter users interacting during #ICPIC2019 were healthcare workers (34%), followed by industry (11%), and academic researchers (8%). These results highlight that Twitter activity during ICPIC2019 scientific congress reached a broader audience than expected. This observation supports the use of Twitter as a communication tool to increase the overall reach of disseminating scientific information [2, 8]. In parallel to other existing commercialized methods to characterize Twitter users and followers (e.g. Symplur healthcare hashtags, Twitonomy), we were able to use this approach to measure the number of distinct followers per user, but at the same time, to keep all followers per user in order to evaluate specific relationships.

The methods used do not only rely on specific words to categorize authors and followers, but rather on their specific frequencies and distributions present in the biographies. These parameters are influenced by multiple factors indicative of gender, culture, personalities and specific interests [22, 23]. Specific interests sometimes converged to provide a clue about professional backgrounds. We observed some clusters to be more specific than others because of the use of a specific lexicon, including healthcare workers and academic researchers. Patient-oriented biographies might include less specific vocabulary and overlap with multiple other categories.

The categories of authors largely influenced categories of followers. This finding has already been observed in a previous study [15]. Furthermore, we observed more diversity in the reach of non-healthcare workers compared to healthcare workers. This observation was also supported by further network analysis between all followers of specific categories. Influencers with a large number of followers might also influence the diversity of reach, impact the reach of Twitter connectedness, and steer conversations [15]. Unfortunately, this information was not accounted for in the analysis.

To note, the population of active followers only represents 0.05 to 0.3% of the total reach. Thus, it should be considered that followers might not always estimate the actual spread of a message. Interestingly, when observing the network of Twitter interactions, different categories of biographies often interacted together. We did not observe particular clusters or over-representation of specific categories, such as healthcare workers in online interactions. In the network analysis, we observed that industries or patients also participated in this online interactions and contributed to the diffusion of conference messages.

Given the homogeneity of Twitter networks from healthcare workers and academics, but the heterogeneity of professions involved in Twitter interactions, the designation of a patient-included™ status and the process of systematically addressing methods to strengthen the inclusion of patients through social media may foster the spread of core messages to non-attending individuals reaching a more diverse population. While this study cannot make this conclusion, Utingen and colleagues performed a social network analysis to analyse Twitter activity from 1672 healthcare conferences and showed that when engaged patients are included in congresses, they increase the spread of conference information flow across social networks [11]. There is little doubt that patient inclusion can have benefits, but identifying the specific advantages requires further attention.

The SARS-CoV-2 pandemic has shifted in-person scientific conferences to virtual and digital events. The shift has provided unprecidented opportunities to use social media platforms including Twitter, to reach a wide audience across the world allowing advanced integration among users and real-time interaction of key findings [25]. Now more than ever it is important to maximize the reach of evidence-based information on infection prevention and control from scientific conferences via social media platforms to debunk misinformation.

## Limitations

First, being unable to confirm participants from the conference from an official list, we only hypothesized that Tweet authors mainly participated in the conference. Second, professions represented in biographies originally represented a mixture of probabilities between different categories. For the sake of simplicity, biographies were categorized only using the most probable category. Therefore, overlapping categories were lost in this analysis (e.g. healthcare worker and academic research). Furthermore, due to the small number of characters allowed for biographies (n = 160), the unsupervised technique is less performant and generalizable. However, above a certain threshold of gamma probabilities, especially considering specific categories, and consistently with the validation of the labels on naive datasets, this technique remained reliable for a majority of biographies. Additionally, this technique accounted for specific distributions of all words included in the biographies to ascertain a category, and not just to specific words. This allowed better discrimination compared to the presence of a single or multiple keywords. Third, only biographies with the most recent tweet composed in English were included, so all other biographies certainly also expressing related professional categories were excluded. Fourth, no other unsupervised or supervised models were performed on the dataset, so repeatability of findings was not assessed. Fifth, we only captured tweets that included the official hashtag of the conference (#ICPIC2019), this might have introduced a selection bias as it is possible that conference-related tweets were sent without the official hashtag [24]. Nonetheless, the use of this performant analysis on a large dataset was able to identify the diversity of biographies from users and followers participating in the online discussion around ICPIC2019. These results add to the body of knowledge on Twitter use from diverse professional background and impact during academic scientific conferences focused on IPC and provide novel insights on the aforementioned points.

## Conclusion

This study offers a unique perspective of the widespread reach of IPC messaging through the use of Twitter social media platform from a single conference. It highlights the potential to increase the dissemination of research across on an array of networks thereby increasing the total Twitter output generated from in-person and virtual scientific conferences. The systematic analysis based on Twitter biographical information can be a useful adjunct to other methods utilised in data science, providing a feasible and useful future direction for the exploration of reach. Furthermore, the present study also suggests that patient-included™ conferences may have a positive impact on overall reach not only to other patients and the public in general, but for the engagement of numerous stakeholders ranging from media to industry, key for IPC. Congress organizers should implement a social media strategy and promote the use of Twitter conference hashtag pre, post and during the event. This strategy offers a useful direction to help disseminate timely information and increase virtual participation of patients, the public and non-attending individuals as highlighted in the patient-includedTM conference charter clauses.

## Supplementary information

**Additional file 1.** **File 1**: Supplementary tables. **File 2**: Definitions. **File 3**: Latent-dirichlet allocation. **File 4**: Supplementary figures. **File 5**: Label and validation of categories. **File 6**: R Code and crude data

## Data Availability

The datasets used and/or analysed during the current study are available from the corresponding author upon request.

## Abbreviations

AMR: Antimicrobial resistance
ICPIC: International Consortium for Prevention and Infection Control
IPC: Infection prevention and control
LDA: Latent Dirichlet allocation
