# Supplementary material for: Enhancing engagement beyond the conference walls: analysis of Twitter use at #ICPIC2019 infection prevention and control conference
Source: Antimicrob Resist Infect Control. 2021 Jan 25;10:20. doi: 10.1186/s13756-021-00891-1 (PMC7830043; doi:10.1186/s13756-021-00891-1)
Supplement: Supplementary file 1 — Additional file 1. File 1: Supplementary tables. File 2: Definitions. File 3: Latent-dirichlet allocation. File 4: Supplementary figures. File 5: Label and validation of categories. File 6: R Code and crude data [file 13756_2021_891_MOESM1_ESM.docx]

**Additional File: Enhancing engagement beyond the conference walls: Analysis of Twitter use at #ICPIC2019 infection prevention and control conference**

Romain Martischang MD^1^#, Ermira Tartari, MSc^1,2,3^#, Claire Kilpatrick, MSc ^4^, Graham Mackenzie MD^5^, Vanessa Carter^6,7^, Enrique Castro-Sánchez PhD^8^, Hilda Márquez-Villarreal MD ^9^, Jonathan A. Otter PhD^8^, Eli Perenchevich MD, MS^10^, Denise Silber^11^, Julie Storr, MHS ^4^, Jason Tetro^12^, Andreas Voss, MD, PhD^13^, Didier Pittet MD, MS, CBE^1^

*^1^ Infection Control Programme and WHO Collaborating Centre on Patient Safety, University of Geneva Hospitals and Faculty of Medicine, Geneva, Switzerland*

*^2^ Institute of Global Health, Faculty of Medicine, University of Geneva, Geneva, Switzerland*

*^3^ Faculty of Health Sciences, University of Malta, Msida, Malta*

*^4^ S3 Global, Glasgow, United Kingdom*

*^5^ NHS Education for Scotland, UK*

*^6^ Healthcare Communications, Social Media, South Africa*

*^7^ Stanford University Medicine X ePatient Scholar Program, Stanford, California*

*^8^ NIHR Health Protection Research Unit (HPRU) in HCAIs and AMR at Imperial College London, and Imperial College Healthcare NHS Trust, Infection Prevention and Control, London, UK*

*^9^ Department of Public Health, University of Guadalajara, Jalisco, Mexico*

*^10^ Center for Access & Delivery Research & Evaluation (CADRE), Iowa City Veterans Affairs Health Care System, Iowa City, IA, USA; Department of Internal Medicine, University of Iowa Carver College of Medicine, Iowa City, IA, USA.*

*^11^ Basil Strategies, Paris, France*

*^12^ Infection Prevention and Control consultant, Edmonton, Alberta, Canada*

*^13^ Department of Medical Microbiology, Radboud University Medical Center, Nijmegen, the Netherlands; Department of Clinical Microbiology and Infectious Diseases, Canisius-Wilhelmina Hospital, Nijmegen, the Netherlands; REshape Center for Innovation, Radboud University Medical Center, Nijmegen, the Netherlands.*

This appendix has been provided by the authors to provide readers additional information about this study.

**Table of contents**

[I. Additional file 1: Supplementary tables 3](#_Toc60475640)

[II. Additional File 2: Definitions 6](#_Toc60475641)

[III. Additional File 3: Latent-Dirichlet Allocation 7](#_Toc60475642)

[IV. Additional file 4: Supplementary figures 8](#_Toc60475643)

[V. Additional File 5: Label & Validation of categories 14](#_Toc60475644)

[VI. R Code and crude data 15](#_Toc60475645)

## **Additional file 1: Supplementary tables**

Supplementary table 1. Patients Included™ conference charter clauses:

1. 1. Patients or caregivers with experience relevant to the conference’s central theme actively participate in the design and planning of the event, including the selection of themes, topics and speakers.
2. 2. Patients or caregivers with experience of the issue addressed by the event participate in its delivery and appear in its physical audience.
3. 3. Travel and accommodation expenses for patients or carers participating in the advertised programme are paid in full, in advance. Scholarships are provided by the conference organisers to allow patients or carers affected by the relevant issues to attend as delegates.
4. 4. The disability requirements of participants are accommodated. All applicable sessions, breakouts, ancillary meetings, and other programme elements are open to patient delegates.
5. 5. Access for virtual participants is facilitated, with free streaming video provided online wherever possible.

Supplementary table 2. Agreement stratified between clusters


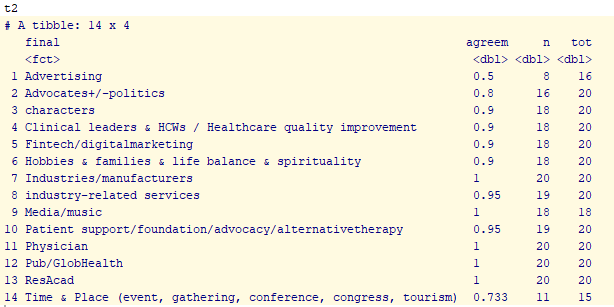


Supplementary table 3. Performance of labels according to gamma categories and topics


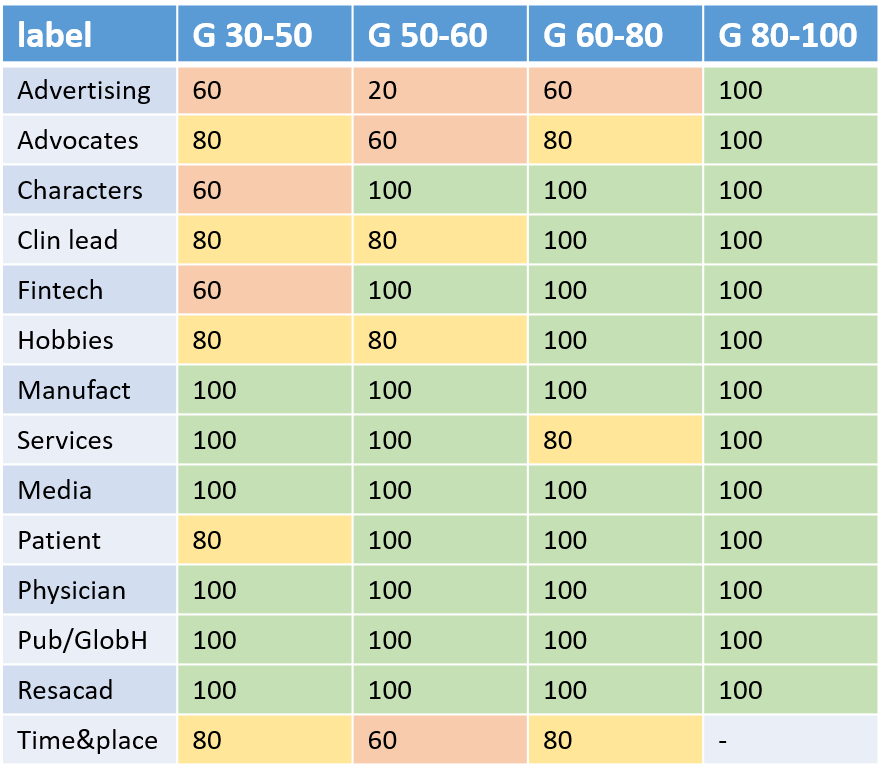


Supplementary table 4. Proportions of clusters among categorized followers with a gamma > 40% and their gamma distribution

| Topic | Sum  (n tot = 181’192) | Proportion | Average gamma proportion (sd) |
| --- | --- | --- | --- |
| Media and music | 5’909 | 3% | 53% (9) |
| Fintech – digital marketing | 11’269 | 6% | 56% (11) |
| Patient-oriented | 8’733 | 5% | 55% (10) |
| Advertising | 6’933 | 4% | 53% (10) |
| Global and public health | 9’559 | 5% | 55% (10) |
| Academic research | 12’449 | 7% | 57% (11) |
| Others | 72’761 | 40% | 61% (13) |
| Industries | 27’690 | 15% | 60% (12) |
| Healthcare workers | 25’889 | 14% | 59% (12) |

Supplementary table 5. Proportions of clusters among categorized authors with a gamma >40% and their gamma distribution

| Topic | Sum  (n tot = 363) | Proportion | Average gamma proportion (sd) |
| --- | --- | --- | --- |
| Media and music | 1 | 0.3% | 51% (-) |
| Fintech – digital marketing | 14 | 4% | 49% (6) |
| Patient-oriented | 5 | 1% | 51% (6) |
| Advertising | 2 | 0.6% | 50% (12) |
| Global and public health | 30 | 8% | 53% (10) |
| Academic research | 29 | 8% | 55% (11) |
| Others | 121 | 33% | 65% (13) |
| Industries | 39 | 11% | 60% (11) |
| Healthcare workers | 122 | 34% | 58% (12) |

Supplementary table 6. Proportions of clusters among followers that actively interacted with authors

| Topic | Sum | Proportion  (among active followers) | Proportion  (among total followers) |
| --- | --- | --- | --- |
| Media and music | 5 | (1.5%) | (0.05%) |
| Fintech – digital marketing | 17 | (5%) | (0.1%) |
| Patient-oriented | 6 | (1.8%) | (0.04%) |
| Advertising | 3 | (1%) | (0.03%) |
| Global and public health | 42 | (12%) | (0.3%) |
| Academic research | 37 | (11%) | (0.2%) |
| Others | 64 | (19%) | (0.07%) |
| Industries | 38 | (11%) | (0.01%) |
| Healthcare workers | 126 | (37%) | (0.04%) |

## **Additional File 2: Definitions**

**Pre-processing:** Exclusion of non-English tweets, and duplicate biographies from different accounts. Words with less than two characters were excluded because they were less likely to be informative of the author’s background, and words expressed more than twice were removed, to avoid the creation of false clusters. Cleaning, stemming, and tokenization process were also part of the pre-processing (explained below). Finally, the resulting matrix was converted into a term-to-document matrix weighted by term frequency (explained below).

**Cleaning process:** Biographies were cleaned by lowering the capital characters, deletion of numbers, specific characters (parenthesis, comas...), stop-words (and, or...), by stemming and tokenization (explained below).

**Stemming process**: rounding the different words to their common root to ensure comparability between documents (for example : preventionist and prevention are both rounded to prevention).

**Tokenization process:** Twitter profile is “exploded” with 1 row per word per biography instead of 1 row per profile.

**Term-to-document matrixes weighed by term frequency:** matrixes organized according to tf–idf (term frequency–inverse document frequency) value. TF-IDF is a statistic that aims to reflect the importance of a word in a document, and of this word in a corpus. It is used as a weighting component to increase efficiency of text mining. The tf–idf value increases proportionally to the frequency of a word in the document and is offset by the number of documents containing this same word in the corpus. This allows to adjust for common, non-specific words.

**Description of Biographies:** Further analysis performed to describe biographies were “bag-of-word” and “bi-gram” analysis.

**Perplexity:**

The perplexity value is used to estimate generalizability of text models across multiple documents. By measuring the normalized log-likelihood of a held-out (validation) dataset, it captures the surprise of models developed using a training dataset when facing new texts. Traditionally, the number of topics selected is a balance between an optimized perplexity and the interpretability of topics. The perplexity might be measured using the formula below:


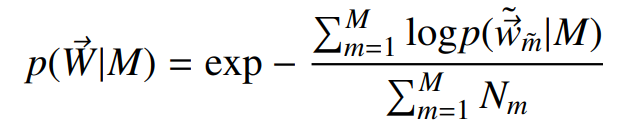


where M is the trained model, w_m_ is the word vector in document m, and N is the number of words in the document m. The lower the perplexity value, the higher the generalizability of distributions measured from the training dataset to other datasets.

## **Additional File 3: Latent-Dirichlet Allocation**

Among all Natural Language Processing methods, the unsupervised clustering method named “Latent Dirichlet Allocation” (LDA) has been used in multiple field to cluster information from social media. This method developed by Prof. David M. Blei in 2003 aims to understand, organize and label unstructured and unknown documents to make informed decisions. As an unsupervised clustering analysis, this method does not need previous labelling of the information. Rather, from naïve texts, this method will estimate a probability of words to belong to a topic (beta distribution), and a probability of topic to belong to a document (gamma distribution). The LDA is based on a specific assumption for the creation of documents: a topic is randomly attributed to a given document from a previously defined number of topics according to a multinomial distribution, and a word is randomly chosen from the attributed topic according to another multinomial distribution. The probability of the document to belong to certain topics is then a hierarchical conditional probability (given a distribution of words). LDA uses therefore Bayesian statistic and Monte Carlo Markov Chains to compute, based on observed biographies, the posterior distribution of these two probabilities (ie: maximizing the likelihood of these probabilities based on observed data).

In this method, only specific steps are controlled by the user: the number of topic to categorize biographies, and the tuning of the two hyper-parameters (alpha and delta). The overall dataset was partitioned between a training and a validation dataset (80 and 20%). The number of topics desired, and the prior probabilities to cluster (alpha and delta proportions) were selected to minimize the perplexity, which is a measure comparing fitted word distribution predicted by the initial model given a topic, with actual word distribution in a naive dataset. For this, a model was performed on the training dataset. Perplexity (detailed above) was calculated when applying this model to the validation dataset for different ranges of values and for each parameter. Once those parameters defined, the model is then developped on a training dataset, including 80% of biographies from the original dataset. In order to apply the developped model on other biographies, words used in the original training dataset are extracted as a vocabulary list. Words from the other biographies that are not part of this list are excluded. Posterior distribution of topics (gamma proportions) from documents are then computed using the same model with the same vocabulary. In order to improve the repeatability of this random process, a seed number was generated. Following the method above, we selected 15 topics, with an alpha at 0.2 and delta at 0.1 (Supplementary figure 5 & 6).

## **Additional file 4: Supplementary figures**

| A  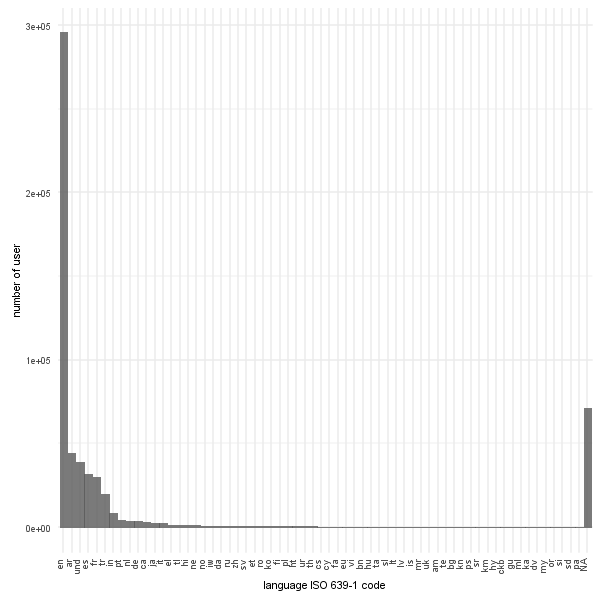 | B  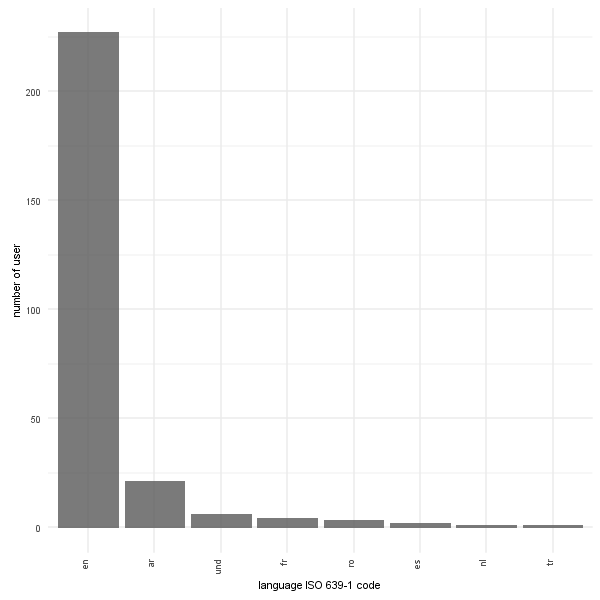 |
| --- | --- |

Supplementary Fig. 1.Counts of language expressed by followers (A) and authors (B)


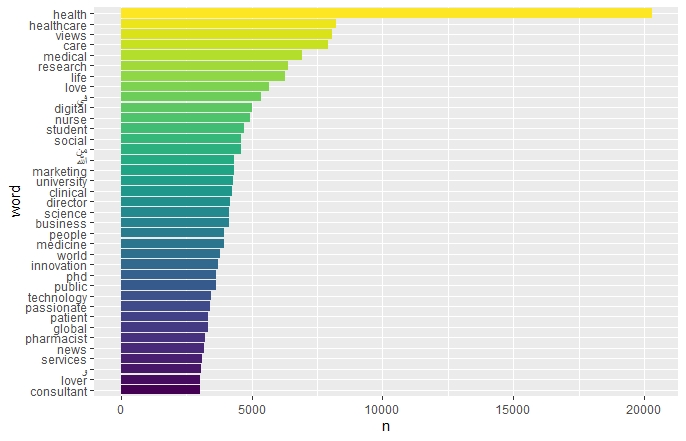


Supplementary Fig. 2. Most recurrent words from the biographies of followers


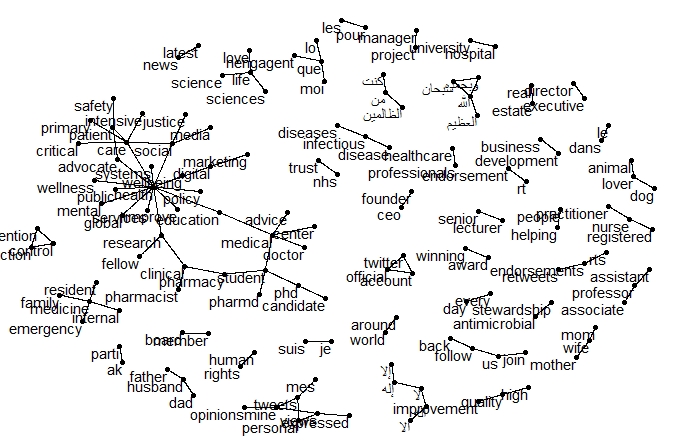


Supplementary Fig. 3. Bi-gram analysis


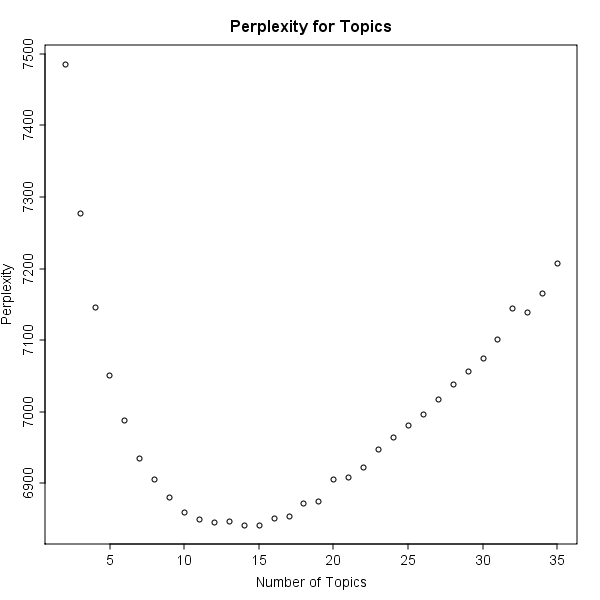


Supplementary Fig. 4. Perplexity of LDA model according to their number of topic


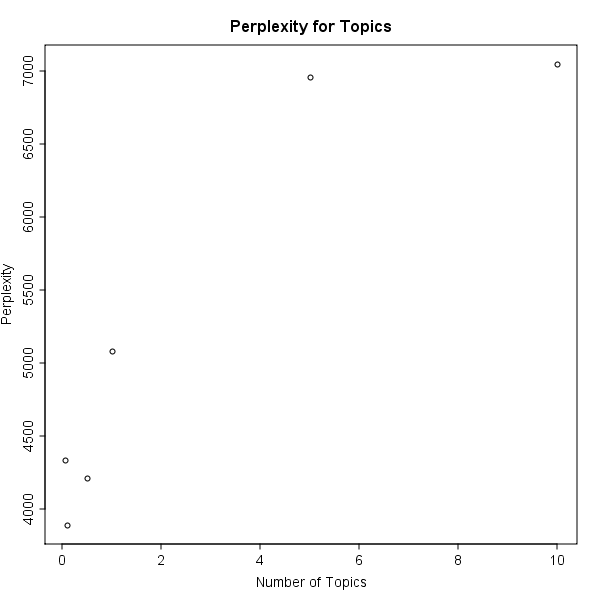


Supplementary Fig. 5. Perplexity for multiple alpha parameters


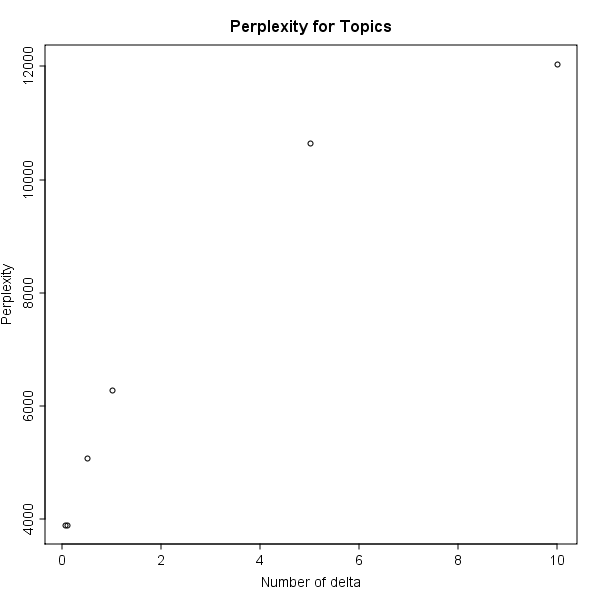


Supplementary Fig. 6. Perplexity for multiple delta parameters


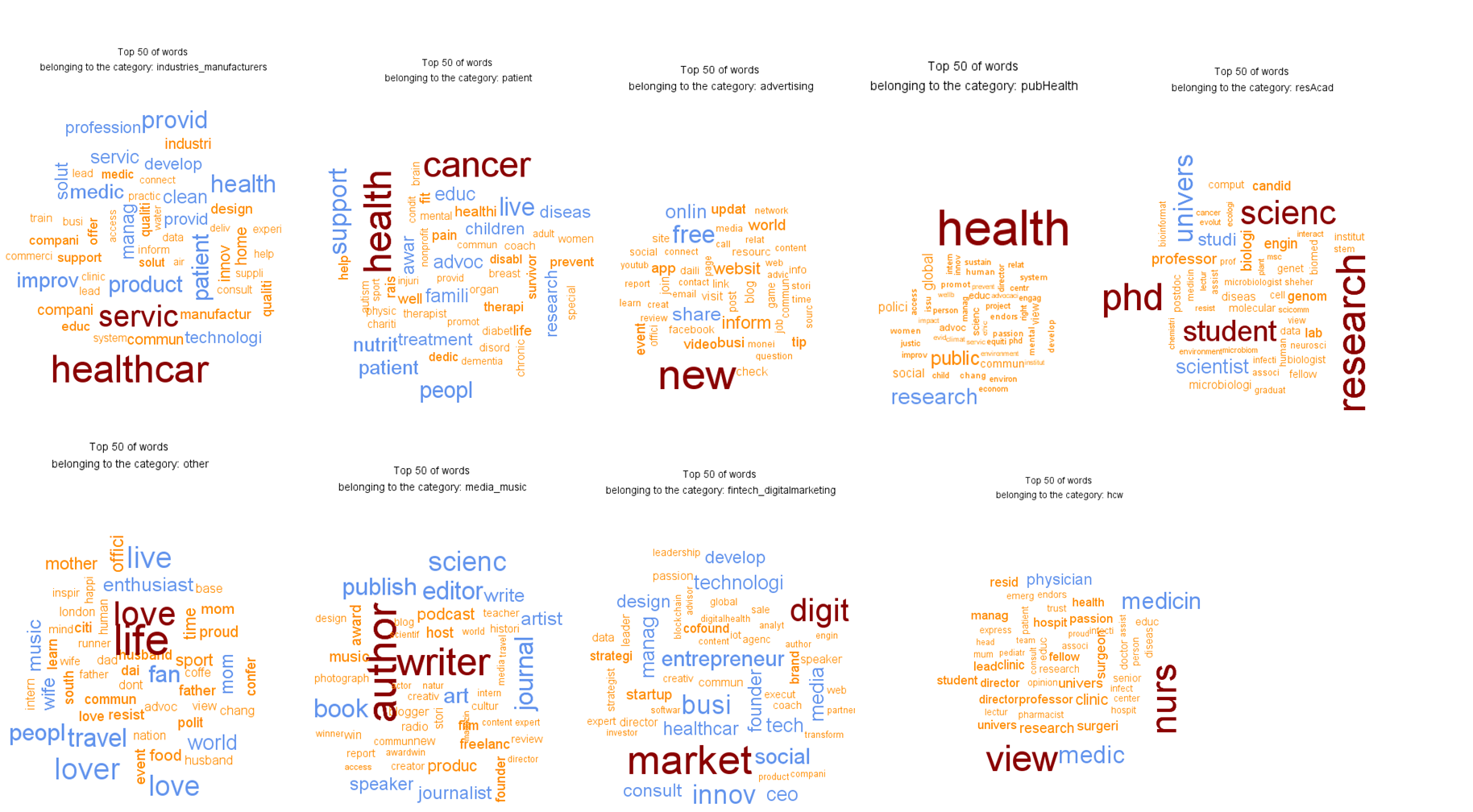


Supplementary Fig. 7. Wordclouds of the top 50 words specific for each cluster


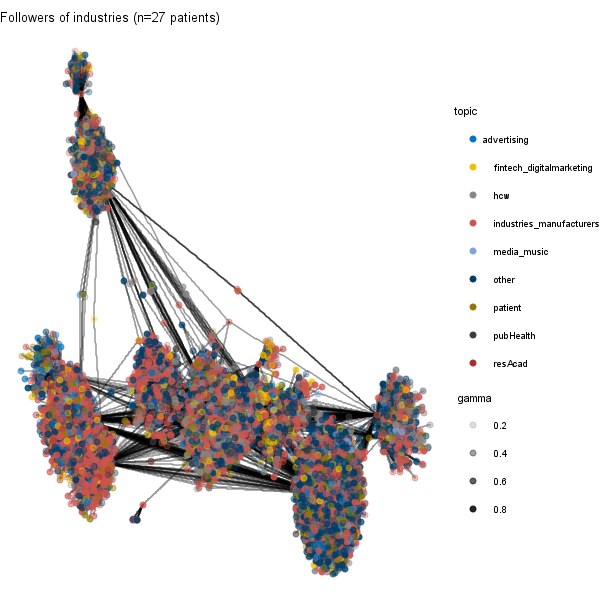


Supplementary Fig. 8. Network analysis of followers from industries (gamma >0.5)


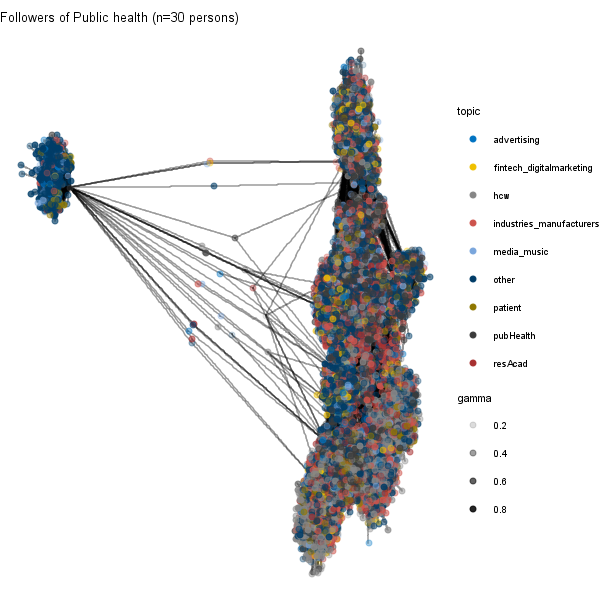


Supplementary Fig. 9. Network analysis of followers from Public and Global health (gamma >0.4)


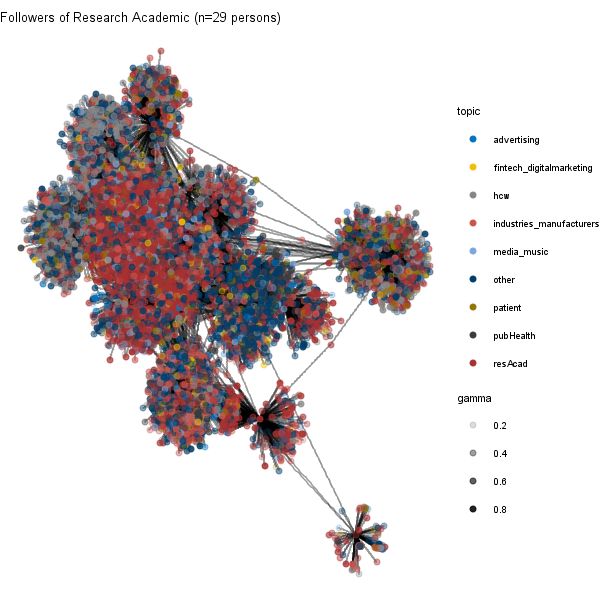


Supplementary Fig. 10. Network analysis of followers from Academic Research (gamma >0.4)


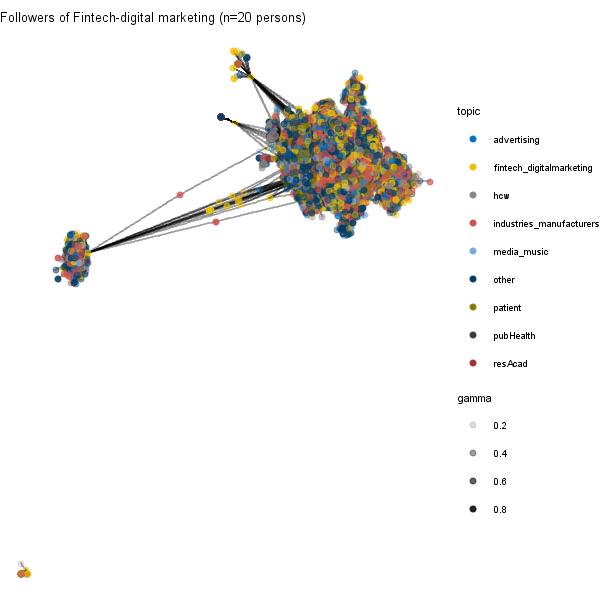


Supplementary Fig. 11. Network analysis of followers from Fintech-Digital marketing (gamma >0.3)

## **Additional File 5: Label & Validation of categories**

15 categories of Twitter biographies were created and investigated. After estimation of gamma and beta probabilities, reviewing of the biographies with the highest probability to belong to each topic, and reviewing of the words most likely associated with each topic, it was necessary to define a label for each cluster. Labels were defined by two blinded researchers (RM and ET) based on the 30 biographies with the highest gamma probability and the 20 words with the highest beta probability for each cluster. For further help, wordclouds of the 50 most frequent words from biographies in each cluster were computed. Similar biographies were then labelled “Advocates and politics”, “Clinical leaders and healthcare workers & healthcare quality improvement”, “Fintech & digital marketing”, “Hobbies, families & life balance”, “Industries and manufacturers”, “Industry related services”, “Media and music”, “Patient support, foundation, advocacy and alternative therapies”, “Physicians”, “Public and global health”, “Academic research”, “Times and places”, and “characters”. Two similar clustered were merged together: “hobbies” and “spirituality”. These 14 labels are detailed in Table 3. For simplification purposes, and considering a low performance for non-pertinent clusters, and a cluster named “other” was created gathering “advocates & politics”, “hobbies and family”, “characters”, and “time and place”. “Clinical leaders and healthcare workers” and “physicians” were merged together, so as “industry-related services” and “manufacturers”, for a total of 9 clusters remaining.

Discordancies were resolved by consensus. These labels were then validated on a naive dataset (not used during the definition of labels), including five documents randomly extracted per four categories of gamma probabilities (30-50;51-60;61-80;81-100%) for all clusters. This even representation of biographies within a range of gamma probabilities helped to define a threshold of gamma proportion to ascertain a topic to a biography. Biographies previously used to define the label were not validated. In case of doubt during the validation of these labels, the professional background of the authors was manually searched through the Internet.

During the validation of these 15 labels, 81.8% of agreement was reached between the two researchers, and overall performance of the label was 90% when assessing the real background of the author (Supplementary table 2). When stratifying the performance according to the category of gamma probability, we reached 100% of performance for a gamma probability between 80 and 100%. When considering a gamma probability of 50-60% of belonging to a cluster, the performance varied among clusters, being acceptable except for advertising, advocates and time & places (Supplementary table 3).

## **R Code and crude data**

Available on demand
